# From hematuria warning to precision immunotherapy: an AI-assisted cross-platform analysis of bladder cancer information on social media

**DOI:** 10.3389/fmed.2026.1910134

**Published:** 2026-09-04

**Authors:** Peng Han, Xin Yang, Qiuxiang Li, Pengcheng Lu, Penghuan Wang, Zhonglei Deng, Zeping Gui, Yiping Zong, Mingyu Liu

**Affiliations:** 1 Department of Urology, The First Affiliated Hospital with Nanjing Medical University, Nanjing, Jiangsu, China; 2 Department of Urology, Jiangsu Key Laboratory of Urological Disease Prevention and Treatment, The Second Affiliated Hospital of Nanjing Medical University, Nanjing Medical University, Nanjing, Jiangsu, China; 3 The Second Clinical Medical College, Nanjing Medical University, Nanjing, Jiangsu, China; 4 Department of Urology, The Affiliated Yixing Hospital of Jiangsu University, Yixing, Jiangsu, China

**Keywords:** artificial intelligence, bladder cancer, hematuria, immunotherapy, precision medicine, social media

## Abstract

**Objective:**

Bladder cancer often presents with hematuria, while modern management increasingly depends on pathology-based risk stratification, immunotherapy, and biomarker-informed treatment. This study evaluated whether social media videos adequately connect early hematuria warning with precision bladder cancer care.

**Methods:**

We conducted a cross-sectional, AI-assisted structured extraction and urologist consensus-scored content analysis of publicly available videos from Bilibili, Douyin, and YouTube. Videos were searched on May 17, 2026, using “血尿” for the hematuria module and “膀胱癌” for the bladder cancer module, with relevance ranking. For each module, one predefined keyword was used for video retrieval to maintain a consistent and reproducible search strategy across platforms. DISCERN, GQS, PEMAT, general content completeness, the Bladder Cancer Warning Score (BCWS), and the Bladder Cancer Precision Immunotherapy Score (BC-PIS) were assessed. After rubric calibration, the three reviewer-level BC-PIS scores were averaged per video for the primary quantitative analysis, and videos with a mean BC-PIS ≥11 were classified as adequate.

**Results:**

In the hematuria module, 368 of 400 videos were included. BCWS differed significantly across platforms, with mean scores of 6.59 ± 1.57 for Bilibili, 7.34 ± 1.09 for Douyin, and 7.99 ± 1.65 for YouTube (*P* < 0.001). Adequate warning information was present in 48/120, 79/99, and 120/149 videos, respectively. In the bladder cancer module, 363 of 395 videos were included. The three-rater mean BC-PIS differed significantly across platforms: 3.28 ± 3.45 for Bilibili, 10.21 ± 2.03 for Douyin, and 7.71 ± 4.75 for YouTube (*P* < 0.001). Adequate precision immunotherapy information was identified in 7/124, 33/90, and 44/149 videos, respectively. Reliability analysis yielded overall ICC(2,1) values of 0.693 for BCWS and 0.876 for BC-PIS; corresponding Fleiss' kappa values were 0.691 and 0.725. The calibrated Douyin reassessment improved BC-PIS reproducibility to an ICC(2,1) of 0.783 (95% CI, 0.653–0.864) and a Fleiss' kappa of 0.598 (95% CI, 0.459–0.718). All 149 included YouTube hematuria videos were matched by platform-specific sequence number and retained in the BCWS reliability analysis.

**Conclusion:**

Social media videos incompletely connect hematuria warning with pathology-based precision immunotherapy in bladder cancer. Greater professional involvement and platform-level quality control are needed to improve online bladder cancer education.

## 1 Introduction

Bladder cancer is one of the most common urological malignancies and remains a major clinical and public health burden (1). Its clinical course is heterogeneous, ranging from non-muscle-invasive disease requiring local management and surveillance to muscle-invasive or metastatic disease requiring multimodal treatment. Early recognition, appropriate diagnostic evaluation, and accurate risk stratification are essential for improving patient outcomes.

Hematuria is one of the most important warning signs of bladder cancer. For many patients, visible or microscopic blood in the urine may be the first symptom that prompts medical attention. However, hematuria can also be caused by benign conditions such as urinary tract infection, urinary stones, benign prostatic disease, trauma, or medication-related bleeding. Because of this broad differential diagnosis, public-facing information should avoid both excessive reassurance and unnecessary fear. Ideally, hematuria education should clearly communicate that hematuria is not always cancer but should prompt timely medical evaluation, particularly when it is painless, gross, recurrent, or unexplained (2).

The diagnosis of bladder cancer depends on a structured clinical pathway that includes urinalysis, imaging, cystoscopy, transurethral resection of bladder tumor (TURBT), and histopathological assessment. Pathology plays a central role in confirming malignancy and determining tumor grade, stage, histological subtype, and the distinction between non-muscle-invasive bladder cancer (NMIBC) and muscle-invasive bladder cancer (MIBC). These pathological findings guide surveillance intensity, intravesical therapy, radical treatment, systemic therapy, and prognosis. Bladder cancer management has also become increasingly connected to immunology and precision oncology. Intravesical bacillus Calmette-Guerin (BCG) remains a key immunotherapeutic strategy for selected patients with NMIB (3). In advanced, metastatic, or selected perioperative settings, systemic immune checkpoint inhibitors targeting PD-1 or PD-L1 have become important therapeutic options (4). In addition, biomarker-informed and targeted treatment strategies, including FGFR-related approaches, are increasingly relevant to individualized bladder cancer care (5). Therefore, high-quality patient education should connect early warning symptoms, diagnostic evaluation, pathological risk stratification, and modern immunotherapy or precision treatment.

Social media platforms have become major sources of health information for the public. Short-video and video-sharing platforms such as Douyin, Bilibili, and YouTube are widely used by patients to obtain information about symptoms, diagnosis, treatment options, and prognosis. These platforms offer accessible and engaging educational formats, but their open and algorithm-driven nature also raises concerns regarding variable quality, incomplete content, commercial promotion, and misinformation (6). In oncology, incomplete or misleading information may influence health-seeking behavior, patient expectations, and understanding of treatment options. Previous studies have evaluated online video quality for various diseases and urological topics, but most have focused on general information quality rather than the continuity of patient-facing information across a clinical care pathway (7, 8). For bladder cancer, this continuity is particularly important: patients may first encounter hematuria-related content before later searching for information on cystoscopy, pathology, BCG, immune checkpoint inhibitors, and precision therapy. Whether social media videos adequately bridge this information chain remains unclear.

We therefore conducted an AI-assisted, cross-platform analysis of bladder cancer information on Bilibili, Douyin, and YouTube, combining structured extraction with consensus scoring by urologists. We specifically examined two connected modules: hematuria warning information and bladder cancer precision immunotherapy information. By combining general quality tools with bladder cancer-specific content scores, we sought to identify platform-level differences, content gaps, and areas requiring improvement in patient-facing digital bladder cancer education.

## 2 Materials and methods

### 2.1 Study design

This was a cross-sectional content analysis of publicly available social media videos. The study was designed as a two-module analysis. The first module evaluated hematuria-related videos as an early warning entry point for bladder cancer. The second module evaluated bladder cancer-related videos focusing on pathology, staging, intravesical immunotherapy, systemic immunotherapy, and precision treatment. Because this study analyzed publicly available online videos and did not involve interaction with human participants or collection of private personal information, formal ethics approval was not required.

### 2.2 Platforms and search strategy

Videos were collected from Bilibili, Douyin, and YouTube. For each module, up to 150 videos were collected from each platform when available. Fewer Douyin videos were retrieved because the available relevance-ranked results under the predefined keyword were limited. For the hematuria module, videos were searched on May 17, 2026, using the keyword “血尿”. For the bladder cancer module, videos were searched on the same date using the keyword “膀胱癌”. Search results were sorted by relevance ranking on each platform.

### 2.3 Eligibility criteria

For the hematuria module, videos were included if they primarily discussed hematuria, blood in urine, urinary bleeding, or related symptoms and were intended for the general public, patients, or health education purposes. Videos were excluded if they were irrelevant, duplicated, inaccessible, non-medical, purely commercial without substantive health information, or lacked interpretable audio, subtitles, or visual text.

For the bladder cancer module, videos were included if they discussed bladder cancer diagnosis, pathology, staging, treatment, BCG, intravesical therapy, systemic immunotherapy, PD-1/PD-L1, targeted therapy, biomarker testing, or precision treatment. Videos were excluded if they were unrelated to bladder cancer, focused only on other diseases, were duplicates, were purely promotional, were inaccessible, or contained no interpretable educational content.

### 2.4 Data extraction

For each video, the following information was extracted: platform, URL, title, uploader name, uploader type, verification status, commercial tendency, video duration, upload date, number of days online, number of followers, views, likes, comments, favorites or collects, shares, and inclusion or exclusion status. Because platform functions differ, unavailable metrics were coded as unavailable rather than zero. For example, YouTube did not provide favorites or shares, and Douyin views were not consistently available. Uploader type was categorized as urologist, other physician or healthcare professional, hospital or medical institution, health science media, commercial medical organization, patient or family member, general user, other, or unclear.

### 2.5 Reviewers and assessment procedure

All eligible videos were assessed by three reviewers, all of whom were urologists. The reviewers first applied the predefined scoring manual independently. After examination of initial disagreement patterns, particularly for short Douyin bladder cancer videos, the reviewers held a structured calibration discussion focused on the interpretation of the scoring anchors; no video-level target score was prescribed. They then independently rescored the Douyin videos without access to one another's ratings. For BC-PIS, the arithmetic mean of the three domain-level ratings was calculated for each video and summed to obtain the primary total score; adequacy was defined as a three-rater mean total ≥11. This aggregation rule was applied consistently across all three platforms. Reviewer-level ratings were retained for reliability analysis, while non-scale descriptive variables were resolved by adjudication when required.

Inter-rater reliability assessment. Reviewer-level records were aligned within each platform by the prespecified sequence number, as the same sequence denoted the same video in all three workbooks. This approach retained all 363 bladder cancer videos and all 368 hematuria videos. The initial retrieval for each module yielded up to 150 videos per platform, from which duplicates and inaccessible links were removed before screening. The two modules were screened independently, and the similar exclusion numbers are coincidental rather than caused by dataset overlap. No ratings were excluded or imputed. Reliability for the Douyin BC-PIS ratings therefore represents post-calibration independent reassessment, whereas the Bilibili and YouTube estimates represent the retained independent ratings under the same scoring rubric.

### 2.6 Quality assessment

The quality and reliability of included videos were assessed using the DISCERN instrument, the Global Quality Score (GQS), and the Patient Education Materials Assessment Tool for audiovisual materials (PEMAT-A/V). DISCERN was used to evaluate the reliability and quality of consumer health information. The GQS was used to assess the overall flow, educational quality, and usefulness of the video. PEMAT was used to evaluate understandability and actionability, expressed as percentage scores.

### 2.7 General content completeness assessment

For both modules, general content completeness was assessed across six dimensions: definition, symptoms or clinical presentation, risk factors or etiology, diagnostic evaluation, management, and follow-up or consultation. Each dimension was scored from 0 to 2, where 0 indicated no relevant content, 0.5 indicated minimal content, 1 indicated partial content, 1.5 indicated mostly complete content, and 2 indicated extensive content.

### 2.8 Bladder cancer warning score

To evaluate whether hematuria-related videos adequately communicated bladder cancer warning information, we developed the Bladder Cancer Warning Score (BCWS). The BCWS included five items: explicit mention of bladder cancer risk, warning regarding painless or gross hematuria, recommendation for medical or urological evaluation, mention of key diagnostic examinations, and avoidance of misleading reassurance. Each item was scored from 0 to 2, yielding a total score from 0 to 10. A BCWS of 7 or higher was defined as adequate bladder cancer warning information.

BCWS development, weighting, and scoring anchors. The five BCWS items were selected *a priori* to represent the minimum patient-facing sequence from recognition of a potentially malignant presentation to appropriate diagnostic escalation, based on the AUA/SUFU hematuria guideline and EAU bladder cancer guidance (2–4). The items cover complementary, non-substitutable functions: risk recognition, identification of a concerning hematuria phenotype, referral or evaluation advice, diagnostic examination information, and avoidance of false reassurance. No empirical evidence was available to justify differential weighting; therefore, each item was assigned the same maximum contribution (2 points), preventing any single clinical message from dominating the score. Item-level anchors were: 0, absent or contradicted; 1, present but vague, incomplete, or lacking clinically useful context; and 2, explicit, substantially complete, and clinically consistent. For the reassurance item, 0 denoted clearly misleading reassurance or advice likely to delay evaluation, 1 denoted ambiguous or insufficiently qualified reassurance, and 2 denoted no misleading reassurance together with appropriate qualification of benign and malignant possibilities.

The BCWS threshold of ≥7/10 was specified before the platform comparisons as a pragmatic content-adequacy threshold. The threshold was selected to identify videos covering most core warning elements and should be considered exploratory rather than a validated clinical cutoff. The threshold was reviewed during pilot scoring by the three urologists for interpretability and consistent application; the pilot was used to clarify anchors, not as a Delphi process or an external validation study. Accordingly, BCWS should be interpreted as a study-specific content-completeness index, and the label “adequate” does not establish viewer comprehension, clinical accuracy in every detail, or improved health-seeking behavior.

### 2.9 Bladder cancer precision immunotherapy score

To evaluate bladder cancer-specific information regarding pathology and immunotherapy, we developed the Bladder Cancer Precision Immunotherapy Score (BC-PIS). The BC-PIS included five domains: pathological diagnosis, staging and risk stratification, intravesical therapy, systemic immunotherapy, and precision or biomarker-informed treatment. Each domain was scored from 0 to 4, yielding a total score from 0 to 20. A BC-PIS of 11 or higher was defined as adequate precision immunotherapy information. The pathological diagnosis domain included TURBT, biopsy, histopathological confirmation, or histological subtype. The staging and risk stratification domain included NMIBC, MIBC, muscle invasion, TNM stage, grade, CIS, or recurrence/progression risk. The intravesical therapy domain included BCG, intravesical chemotherapy, bladder instillation, or recurrence prevention. The systemic immunotherapy domain included immune checkpoint inhibitors, PD-1, PD-L1, pembrolizumab, nivolumab, avelumab, atezolizumab, or related concepts. The precision treatment domain included FGFR, biomarker testing, molecular testing, targeted therapy, or treatment selection based on pathology, stage, or molecular features.

BC-PIS development, weighting, and scoring anchors. The five domains were defined *a priori* from the clinical decision pathway described in EAU guidance and pivotal evidence for immune checkpoint and FGFR-directed treatment (3–6): pathological confirmation; stage and risk stratification; intravesical treatment; systemic immunotherapy; and biomarker-informed or precision treatment. These domains were retained because each changes the interpretation or applicability of treatment information. Equal domain weighting (0–4 points each) was chosen because there was no validated evidence-based basis for assigning relative weights and because unequal weights could make a high score achievable through extensive discussion of only one treatment component. For every domain, the common anchors were: 0, absent, incorrect, or materially misleading; 1, only named or mentioned without explanation; 2, a basic accurate explanation with limited indication or context; 3, a substantially complete explanation linking the concept to disease setting, patient selection, or treatment purpose; and 4, a comprehensive and clinically contextualized explanation that also addressed relevant indications, limitations, or relationships with pathology, stage, risk, or biomarkers. Reviewers applied these anchors to the domain-specific concepts listed above and resolved borderline cases by consensus.

The BC-PIS threshold of ≥11/20 was prespecified as an operational content-adequacy threshold, not as a clinically validated cutoff. The three-urologist pilot review assessed the clarity and feasibility of applying the anchors but did not estimate construct validity, predictive validity, or generalizability. Thus, BC-PIS ranks the completeness of predefined information in this sample and should not be interpreted as a validated measure of overall video quality or clinical benefit.

### 2.10 AI-assisted structured extraction and urologist consensus scoring

This study used an AI-assisted structured extraction workflow followed by final consensus scoring by urologists. Three generative AI tools, ChatGPT (GPT-5.5, OpenAI), Gemini (Gemini 3.6 Flash, Google), and DeepSeek (DeepSeek-V3), were used to assist in extracting predefined content elements from video titles, descriptions, subtitles, transcripts, and visible on-screen text when available. A predefined structured prompt was applied consistently across AI tools to guide information extraction. When complete transcripts were unavailable, relevant information was extracted from available video materials and subsequently reviewed by investigators.

The extracted elements included hematuria, bladder cancer risk, painless or gross hematuria, medical evaluation, cystoscopy, TURBT, pathology, NMIBC/MIBC, BCG, PD-1/PD-L1, immune checkpoint inhibitors, FGFR, biomarker testing, precision treatment, and potential misinformation.

As most included videos were in Chinese, AI-assisted extraction was performed directly on original-language content, followed by review by urologists to ensure contextual accuracy. AI-generated outputs were used only as preliminary references for structured content review and were not treated as final scores. Three urologists independently reviewed the video information and AI-assisted extraction results according to predefined criteria. For BC-PIS, reviewer-level scores were retained and the three-rater mean was used for quantitative platform comparisons; disagreements in non-scale coding were resolved through discussion. Because the AI tools served only as assistive extraction tools, the study did not treat them as independent evaluators or calculate AI-human agreement.

### 2.11 Statistical analysis

SPSS software version 26.0 was used for all statistical analyses. Categorical variables were presented as frequencies and percentages. Continuous variables were assessed for distribution characteristics before analysis. Normally distributed variables were presented as mean ± standard deviation (SD), whereas skewed variables were presented as median and interquartile range (IQR). Social media engagement metrics, including video duration, views, likes, and comments, were summarized using median (IQR) due to their potentially skewed distributions. Kruskal–Wallis tests were used to compare continuous variables across the three platforms. Chi-square tests or Fisher exact tests were used to compare categorical variables, as appropriate. Spearman correlation analyses were conducted to evaluate associations between DISCERN, GQS, engagement metrics, PEMAT scores, general content completeness scores, BCWS, and BC-PIS. Multiple comparisons were adjusted using the Bonferroni correction when applicable. A two-sided *P*-value < 0.05 was considered statistically significant.

Uploader-type analyses were conducted separately for the hematuria and bladder cancer modules. Prespecified uploader categories with sparse counts were consolidated into urologists, other healthcare professionals, hospitals or medical institutions, health science media, non-professional uploaders (general users and patients or family members), and other/commercial/unclear sources. The per-video mean BCWS and BC-PIS scores derived from the three reviewers were used as the dependent variables. Associations were estimated using separate multivariable linear regression models including uploader type and platform as categorical fixed effects; non-professional uploaders and Bilibili were the reference categories. Adjusted mean differences with 95% confidence intervals were reported. Unadjusted score means and proportions meeting the exploratory BCWS ≥7 and BC-PIS ≥11 thresholds were also summarized. Because several uploader-by-platform cells were sparse or empty, interaction and extensive pairwise analyses were not performed.

For reliability analysis, continuous BCWS and BC-PIS total scores were evaluated using a two-way random-effects, absolute-agreement, single-measure intraclass correlation coefficient [ICC(2,1)] (9). Adequate versus inadequate classifications (BCWS ≥7 and reviewer-specific BC-PIS ≥11) were evaluated using Fleiss' kappa for three raters (10). Estimates are reported with 95% confidence intervals from 5,000 video-level nonparametric bootstrap resamples using fixed random seeds. Reliability was estimated overall and separately by platform. For primary BC-PIS platform comparisons, each video's three reviewer domain scores were averaged before summation; Kruskal–Wallis tests were applied to these per-video mean totals and domains.

## 3 Results

### 3.1 Video selection

**Figure 1:**
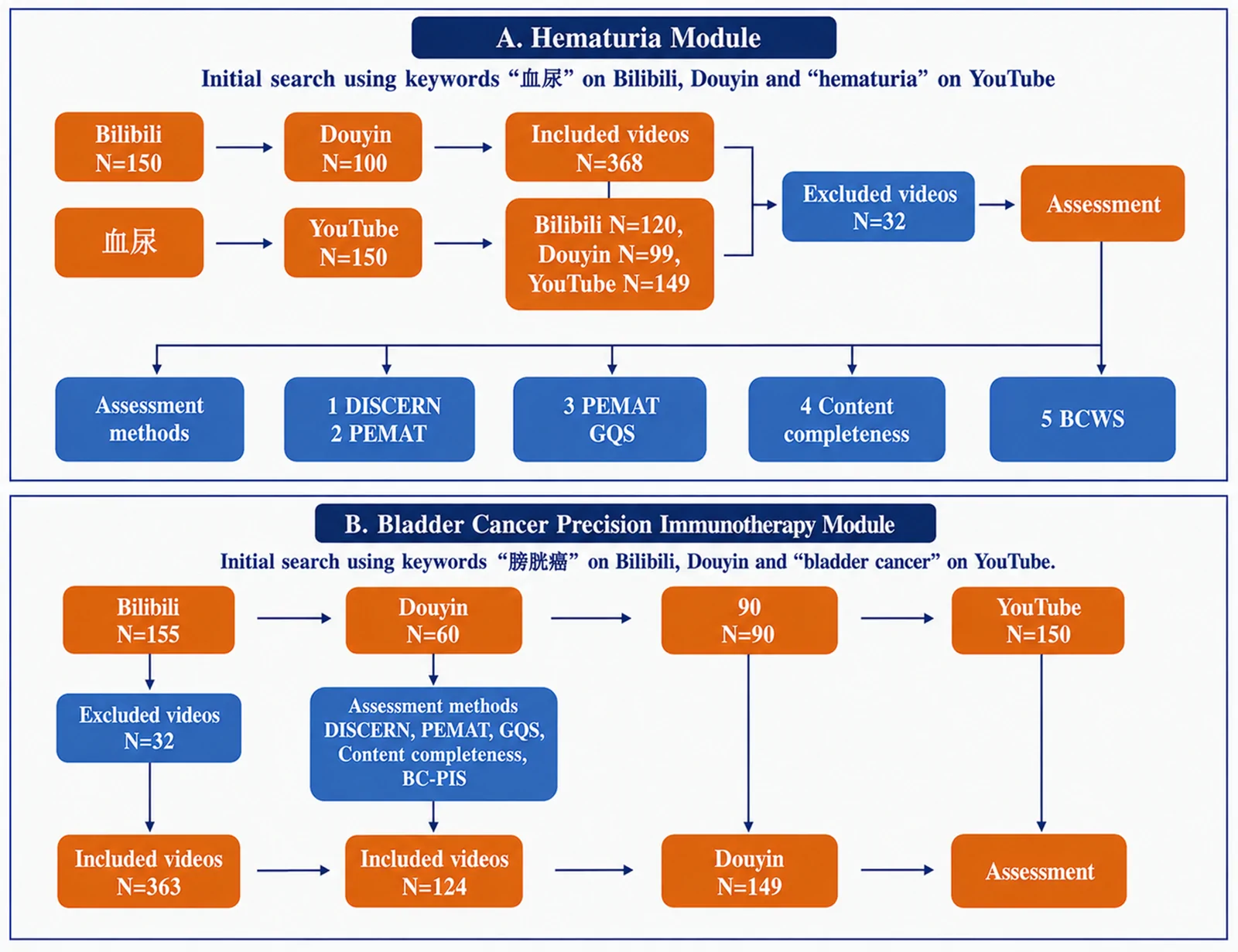
Flowchart of video selection and assessment procedures. The two panels display the parallel workflow used for the **(A)** hematuria-warning and **(B)** bladder-cancer precision-immunotherapy modules; exact platform counts are reported in Table 1.

In the hematuria module, 400 videos were initially retrieved, including 150 from Bilibili, 100 from Douyin, and 150 from YouTube. After screening, 368 videos were included: 120 from Bilibili, 99 from Douyin, and 149 from YouTube (Table 1, Figure 1). In the bladder cancer precision immunotherapy module, 395 videos were initially retrieved, including 155 from Bilibili, 90 from Douyin, and 150 from YouTube. After screening, 363 videos were included: 124 from Bilibili, 90 from Douyin, and 149 from YouTube (Table 1, Figure 1). Figure 1 complements Table 1 by displaying the two analysis modules as parallel selection and assessment workflows, thereby clarifying that the same platform-level procedure was applied to distinct clinical information pathways.

**Table 1:** Video selection and inclusion results.

Module	Platform	Initial records	Included videos	Excluded videos	Inclusion rate
Hematuria warning	Bilibili	150	120	30	80.0%
Hematuria warning	Douyin	100	99	1	99.0%
Hematuria warning	YouTube	150	149	1	99.3%
Hematuria warning	Total	400	368	32	92.0%
Bladder cancer precision immunotherapy	Bilibili	155	124	31	80.0%
Bladder cancer precision immunotherapy	Douyin	90	90	0	100.0%
Bladder cancer precision immunotherapy	YouTube	150	149	1	99.3%
Bladder cancer precision immunotherapy	Total	395	363	32	91.9%

Values are based on the final cleaned datasets used for manuscript analysis. The alternating module shading separates the hematuria-warning and bladder-cancer precision-immunotherapy samples.

### 3.2 General features and quality of hematuria-related videos

Among the 368 included hematuria-related videos, the source of upload differed across platforms. On Bilibili, videos were most frequently uploaded by other physicians or healthcare professionals (44/120, 36.7%), general users (35/120, 29.2%), and urologists (25/120, 20.8%). On Douyin, videos were mainly uploaded by urologists (51/99, 51.5%) and other physicians or healthcare professionals (43/99, 43.4%). On YouTube, health science media (43/149, 28.9%), hospitals or medical institutions (39/149, 26.2%), and urologists (28/149, 18.8%) were the most common sources. The median duration was 177.5 s (IQR, 82.5–407.5) for Bilibili, 77.5 s (IQR, 49.0–131.5) for Douyin, and 370.0 s (IQR, 174.0–804.0) for YouTube (*P* < 0.001; Table 2).

**Table 2:** Quality characteristics of hematuria-related videos by platform.

Variable	Bilibili	Douyin	YouTube	*P*-value
Included videos, n	120	99	149	-
Duration, s	177.5 (82.5–407.5)	77.5 (49.0–131.5)	370.0 (174.0–804.0)	< 0.001
DISCERN total	40.05 ± 7.40	52.90 ± 1.92	50.42 ± 13.01	< 0.001
GQS	2.78 ± 0.96	4.04 ± 0.65	3.56 ± 0.99	< 0.001
PEMAT understandability, %	73.90 ± 23.34	96.50 ± 5.19	64.63 ± 22.44	< 0.001
PEMAT actionability, %	49.17 ± 35.93	91.67 ± 11.85	57.05 ± 33.90	< 0.001
Content completeness	6.03 ± 2.25	9.69 ± 1.07	8.16 ± 2.32	< 0.001
BCWS	6.59 ± 1.57	7.34 ± 1.09	7.99 ± 1.65	< 0.001
Adequate BCWS, n/N (%)	48/120 (40.0%)	79/99 (79.8%)	120/149 (80.5%)	< 0.001

Continuous variables are presented as mean ± standard deviation (SD) for normally distributed variables and median (interquartile range, IQR) for skewed variables. *P* values were adjusted using Bonferroni correction for multiple comparisons. BCWS ≥ 7 was defined as adequate bladder cancer warning information. GQS, global quality score; PEMAT, patient education materials assessment tool; BCWS, bladder cancer warning score. Rows are visually grouped as sample characteristics, general quality metrics, and study-specific content outcomes.

Significant platform-level differences remained after Bonferroni correction in all major quality indicators (Table 2). The mean DISCERN total score was 40.05 ± 7.40 for Bilibili, 52.90 ± 1.92 for Douyin, and 50.42 ± 13.01 for YouTube (*P* < 0.001). The mean GQS score was 2.78 ± 0.96 for Bilibili, 4.04 ± 0.65 for Douyin, and 3.56 ± 0.99 for YouTube (*P* < 0.001). PEMAT understandability was highest on Douyin (96.50 ± 5.19%), followed by Bilibili (73.90 ± 23.34%) and YouTube (64.63 ± 22.44%) (*P* < 0.001). PEMAT actionability was also highest on Douyin (91.67 ± 11.85%), compared with YouTube (57.05 ± 33.90%) and Bilibili (49.17 ± 35.93%) (*P* < 0.001).

### 3.3 Bladder cancer warning information in hematuria videos

**Figure 2:**
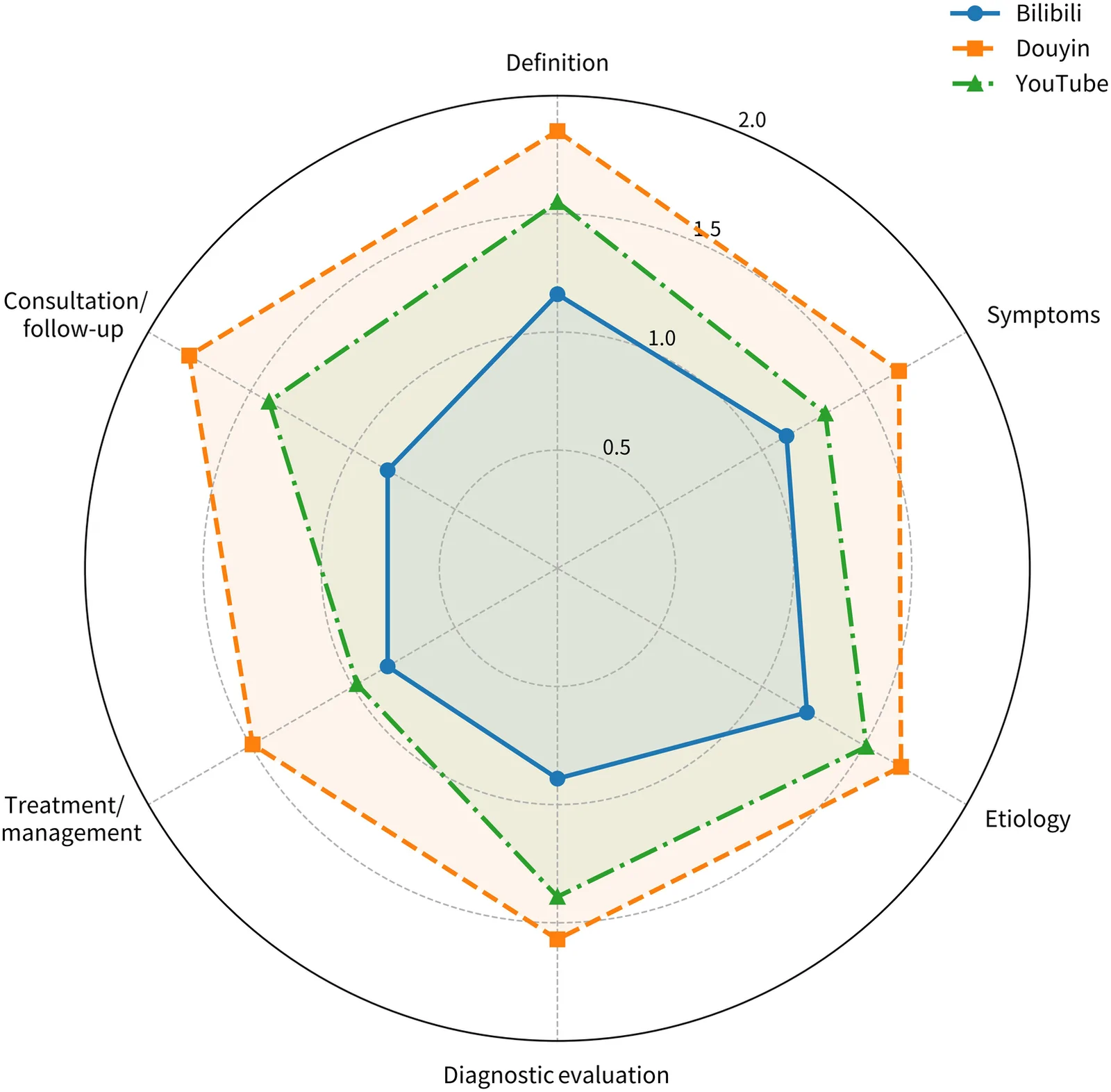
Content completeness of hematuria-related videos. The radar profiles display the relative balance of six educational content domains on a common 0–2 scale. Unlike the aggregate values in Table 2, the figure shows whether a platform's overall score reflects broad coverage or strength in only selected domains.

The mean content completeness score in the hematuria module was 6.03 ± 2.25 for Bilibili, 9.69 ± 1.07 for Douyin, and 8.16 ± 2.32 for YouTube (*P* < 0.001; Table 2). In the six general content domains, hematuria definition, symptoms, etiology, diagnostic evaluation, treatment or management, and consultation or follow-up all differed significantly across platforms (Figure 2). Figure 2 adds a domain-level profile to the aggregate completeness scores in Table 2, showing whether platform differences were distributed across the educational pathway or concentrated in selected content dimensions.

**Figure 3:**
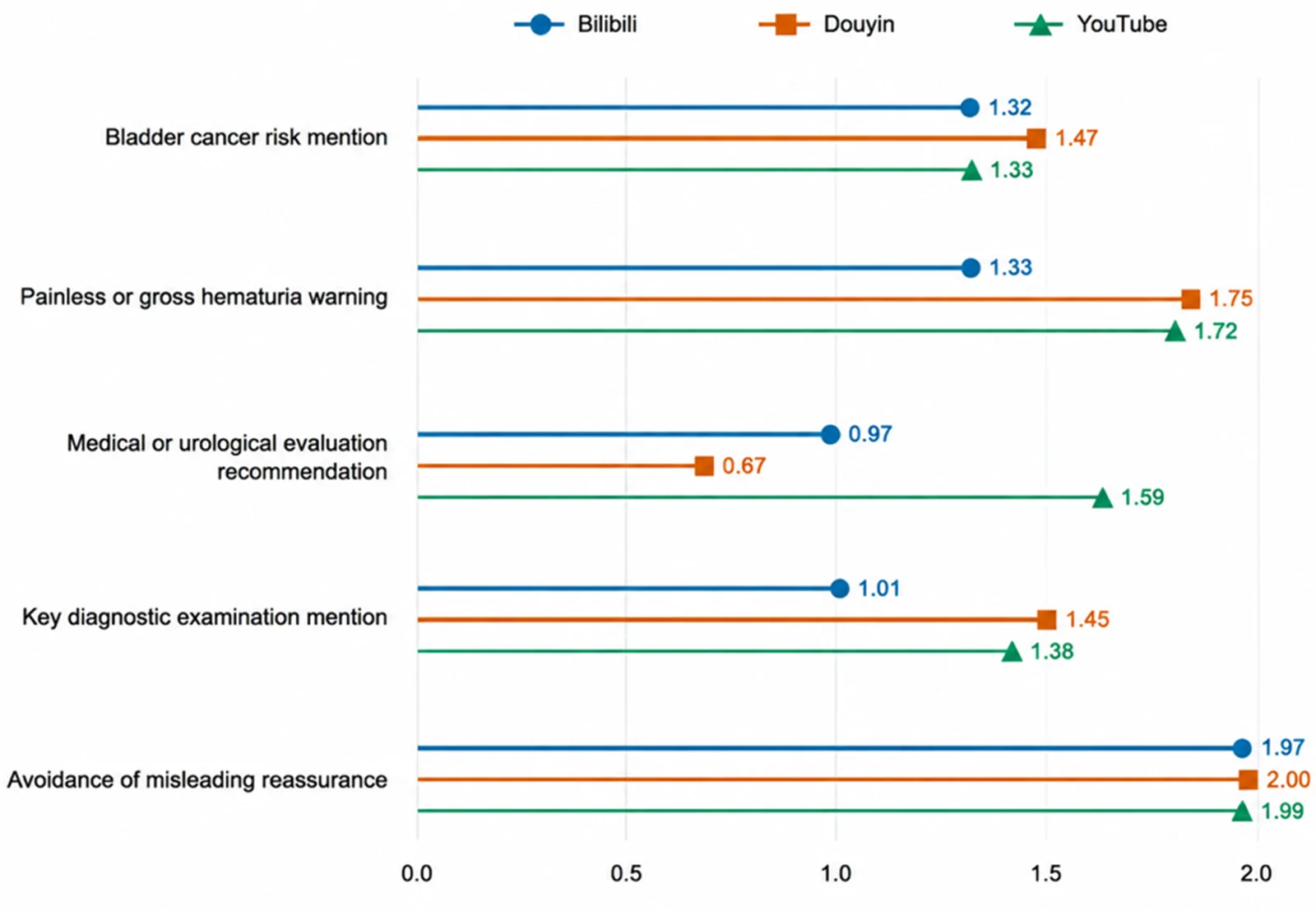
Platform-specific profiles across the five bladder cancer warning score domains. Points and adjacent labels represent platform-level mean scores; each domain ranges from 0 to 2, with higher values indicating more complete warning information. The figure complements Table 3 by showing that platform differences were concentrated in diagnostic escalation messages, while avoidance of misleading reassurance was consistently high.

The mean BCWS was 6.59 ± 1.57 for Bilibili, 7.34 ± 1.09 for Douyin, and 7.99 ± 1.65 for YouTube (*P* < 0.001; Table 2). Adequate bladder cancer warning information, defined as BCWS ≥ 7, was present in 48/120 Bilibili videos, 79/99 Douyin videos, and 120/149 YouTube videos (Table 2). In the five BCWS domains, YouTube and Douyin generally demonstrated more complete bladder cancer warning and diagnostic escalation information than Bilibili, especially regarding painless/gross hematuria warning, medical or urological evaluation recommendation, and key diagnostic examination mention (Table 3, Figure 3). These findings indicate that hematuria-related videos generally avoided overtly misleading reassurance, but the completeness of cancer warning and diagnostic escalation differed substantially across platforms. The Figure 3 makes the source of the total-score differences more explicit: variation was greatest for recommendations concerning medical or urological evaluation, whereas all three platforms scored close to the maximum for avoiding misleading reassurance.

**Table 3:** Bladder cancer warning score domains in hematuria-related videos.

BCWS domain	Bilibili	Douyin	YouTube	Overall	*P*-value
Bladder cancer risk mention	1.32	1.47	1.33	1.37	0.157
Painless/gross hematuria warning	1.33	1.75	1.72	1.6	<0.001
Medical/urological evaluation recommendation	0.97	0.67	1.59	1.14	<0.001
Key diagnostic examination mention	1.01	1.45	1.38	1.28	<0.001
Avoidance of misleading reassurance	1.97	2	1.99	1.99	0.381
Total BCWS	6.59 ± 1.57	7.34 ± 1.09	7.99 ± 1.65	—	<0.001

BCWS domain scores range from 0 to 2, with higher scores indicating more complete bladder cancer warning information. Total BCWS ranges from 0 to 10. Continuous variables are presented as mean or mean ± standard deviation, as appropriate. BCWS, bladder cancer warning score. *P* values were adjusted using Bonferroni correction for multiple comparisons. Table 3 provides the exact domain means, while Figure 3 emphasizes the cross-domain platform profiles.

### 3.4 General features and quality of bladder cancer-related videos

In the bladder cancer precision immunotherapy module, uploader sources differed significantly across platforms. Bilibili videos were most commonly uploaded by urologists (74/124, 59.7%). Douyin videos were primarily uploaded by urologists (63/90, 70.0%) and other physicians or healthcare professionals (25/90, 27.8%). YouTube videos were mainly uploaded by hospitals or medical institutions (68/149, 45.6%), health science media (23/149, 15.4%), and other sources (22/149, 14.8%).

Video duration differed significantly across platforms. YouTube videos were the longest, with a median duration of 289.0 s (IQR, 137.0–853.0), compared with 94.0 s (IQR, 56.75–229.0) on Bilibili and 93.0 s (IQR, 63.75–166.25) on Douyin (*P* < 0.001; Table 4).

**Table 4:** Quality characteristics of bladder cancer-related videos by platform.

Variable	Bilibili	Douyin	YouTube	*P*-value
Included videos, n	124	90	149	-
Duration, s	94.0 (56.75–229.0)	93.0 (63.75–166.25)	289.0 (137.0–853.0)	<0.001
DISCERN total	30.94 ± 6.34	59.28 ± 1.94	42.31 ± 11.57	<0.001
GQS	1.86 ± 1.01	4.20 ± 0.56	3.23 ± 0.96	<0.001
PEMAT understandability, %	78.37 ± 20.66	100.00 ± 0.00	58.33 ± 25.57	<0.001
PEMAT actionability, %	43.21 ± 29.76	100.00 ± 0.00	45.13 ± 30.57	<0.001
BC-PIS	3.28 ± 3.45	10.21 ± 2.03	7.71 ± 4.75	<0.001
Adequate BC-PIS, n/N (%)	7/124 (5.6%)	33/90 (36.7%)	44/149 (29.5%)	<0.001

Continuous variables are presented as mean ± standard deviation (SD) for normally distributed variables and median (interquartile range, IQR) for skewed variables. *P* values were adjusted using Bonferroni correction for multiple comparisons. BC-PIS values and adequacy classifications are based on the per-video mean of three reviewer scores; BC-PIS ≥11 was defined as adequate precision immunotherapy information. GQS, global quality score; PEMAT, patient education materials assessment tool; BC-PIS, bladder cancer precision immunotherapy score. Rows are visually grouped as sample characteristics, general quality metrics, and the primary BC-PIS outcomes.

Bladder cancer-related video quality differed significantly across platforms (Table 4). The mean DISCERN total score was 30.94 ± 6.34 for Bilibili, 59.28 ± 1.94 for Douyin, and 42.31 ± 11.57 for YouTube (*P* < 0.001). The mean GQS score was 1.86 ± 1.01 for Bilibili, 4.20 ± 0.56 for Douyin, and 3.23 ± 0.96 for YouTube (*P* < 0.001). PEMAT understandability was 78.37 ± 20.66% for Bilibili, 100.00 ± 0.00% for Douyin, and 58.33 ± 25.57% for YouTube (*P* < 0.001). PEMAT actionability was 43.21 ± 29.76% for Bilibili, 100.00 ± 0.00% for Douyin, and 45.13 ± 30.57% for YouTube (*P* < 0.001).

### 3.5 Precision immunotherapy information in bladder cancer videos

The three-rater mean BC-PIS differed significantly across platforms. Mean scores were 3.28 ± 3.45 for Bilibili, 10.21 ± 2.03 for Douyin, and 7.71 ± 4.75 for YouTube (*P* < 0.001; Table 4). Adequate precision immunotherapy information, defined as a mean BC-PIS ≥11, was present in 7/124 Bilibili videos, 33/90 Douyin videos, and 44/149 YouTube videos (*P* < 0.001; Table 4).

**Figure 4:**
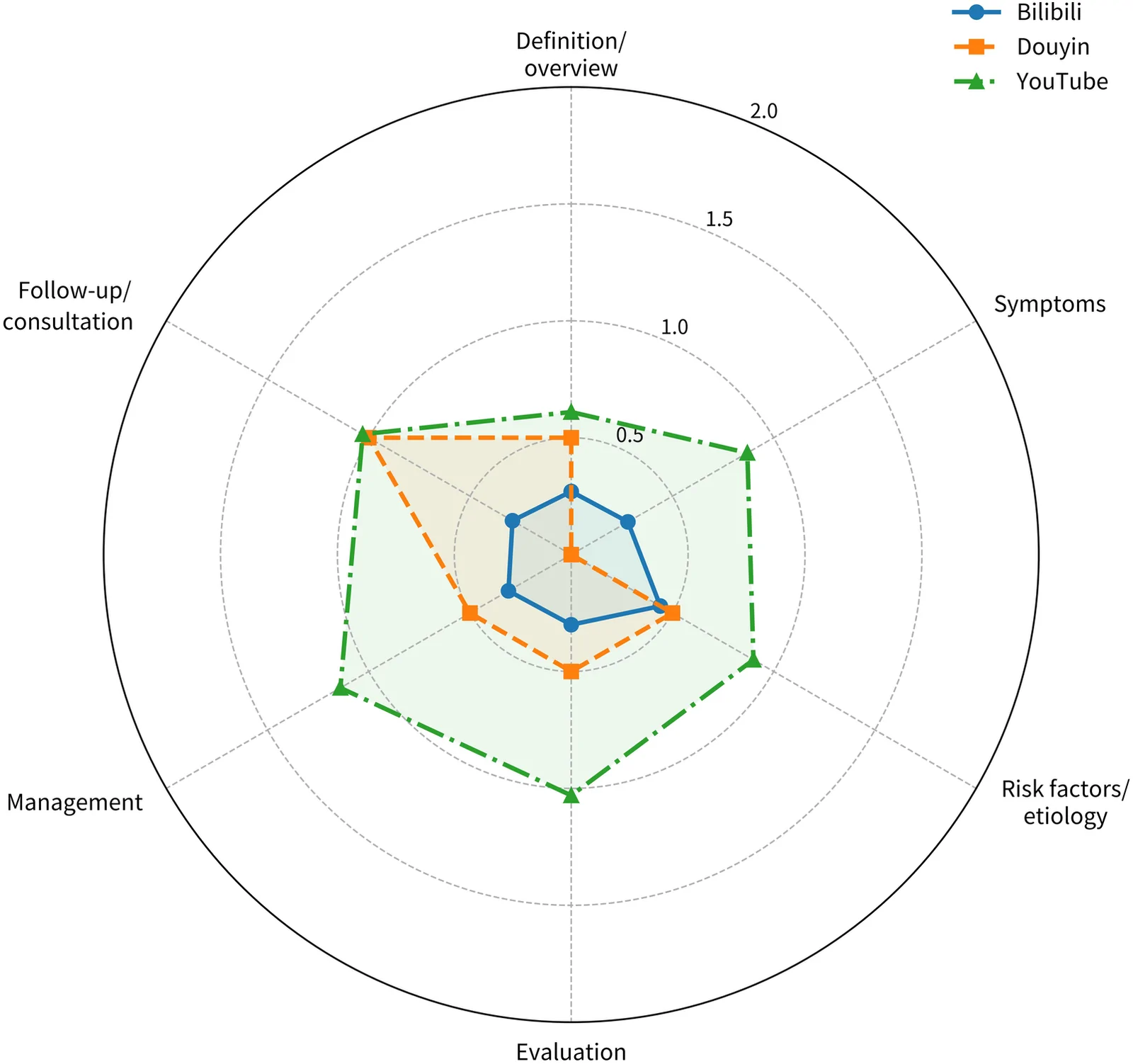
Content completeness of bladder cancer-related videos. The radar profiles display the relative balance of six general content domains on a common 0–2 scale and complement the aggregate quality indicators by showing the configuration of educational coverage within each platform.

**Figure 5:**
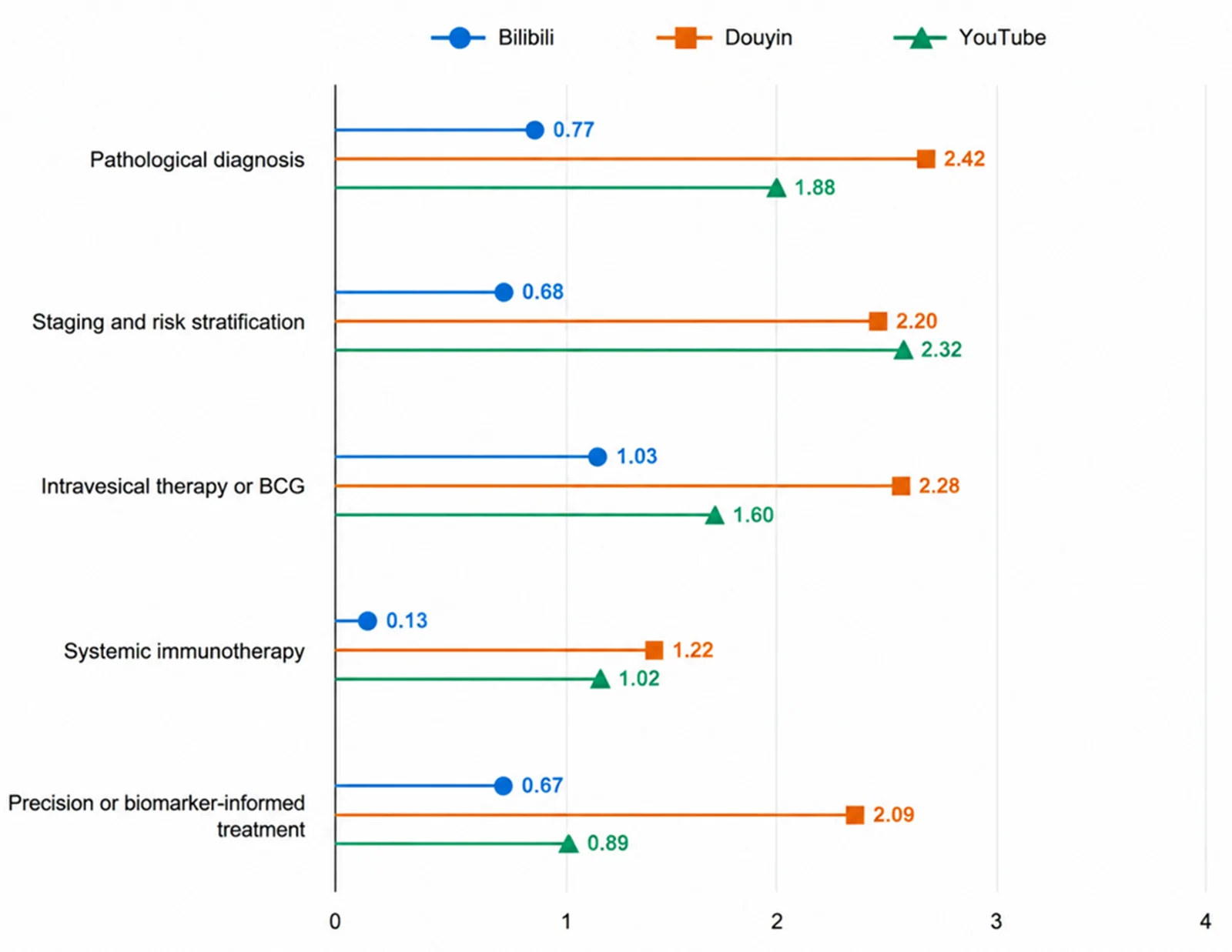
Platform-specific profiles across the five bladder cancer precision immunotherapy score domains. Points and adjacent labels represent platform-level means of the per-video three-rater scores; each domain ranges from 0 to 4. The figure complements Table 5 by showing the balance of coverage across the pathology-to-treatment pathway: Douyin had higher means in four domains, YouTube had the highest staging and risk-stratification score, and Bilibili showed consistently limited coverage, especially for systemic immunotherapy.

Across the five BC-PIS domains, Douyin had the highest mean scores for pathological diagnosis, intravesical therapy, systemic immunotherapy, and precision or biomarker-informed treatment, whereas YouTube had the highest staging and risk-stratification score. YouTube remained higher than Bilibili across all five domains. All domain-level differences were significant (*P* < 0.001; Table 5 and Figure 5). General content completeness also differed across platforms (Figure 4). Figures 4, 5 provide a domain-level view that is not apparent from the total scores alone. Figure 4 shows the balance of general educational coverage, whereas Figure 5 shows that higher total BC-PIS values did not imply uniformly complete coverage of pathology, staging, intravesical treatment, systemic immunotherapy, and biomarker-informed treatment.

**Table 5:** Bladder cancer precision immunotherapy score domains in bladder cancer-related videos.

BC-PIS domain	Bilibili	Douyin	YouTube	*P*-value
Pathological diagnosis	0.77	2.42	1.88	<0.001
Staging and risk stratification	0.68	2.20	2.32	<0.001
Intravesical therapy/BCG	1.03	2.28	1.60	<0.001
Systemic immunotherapy	0.13	1.22	1.02	<0.001
Precision/biomarker-informed treatment	0.67	2.09	0.89	<0.001
Total BC-PIS	3.28 ± 3.45	10.21 ± 2.03	7.71 ± 4.75	<0.001
BC-PIS ≥ 11, n/N (%)	7/124 (5.6%)	33/90 (36.7%)	44/149 (29.5%)	<0.001

Domain scores range from 0 to 4 and are presented as per-video three-rater means aggregated within platform. Lower scores indicate limited or absent information. Total scores are mean ± standard deviation. *P* values were adjusted using Bonferroni correction for multiple comparisons. Table 5 provides the exact domain means, while Figure 5 emphasizes the balance and omissions across the pathology-to-treatment pathway.

Systemic immunotherapy and biomarker-informed precision treatment remained incompletely represented, particularly on Bilibili and YouTube. Mean systemic immunotherapy scores were 0.13 for Bilibili, 1.22 for Douyin, and 1.02 for YouTube (*P* < 0.001; Table 5). Mean precision or biomarker-informed treatment scores were 0.67, 2.09, and 0.89, respectively (*P* < 0.001). The recalibrated estimates indicate that Douyin performed better than the other platforms in several domains but did not provide uniformly adequate precision-immunotherapy information.

### 3.6 Correlation analyses

Spearman correlation analyses showed that DISCERN total score was positively correlated with GQS across platforms. In the hematuria module, videos with higher DISCERN scores generally had better GQS, PEMAT scores, content completeness, and BCWS. Engagement metrics were not consistently correlated with DISCERN or GQS, suggesting that video popularity did not reliably reflect medical information quality. In the bladder cancer module, DISCERN scores were positively associated with GQS and BC-PIS on Bilibili, while engagement metrics showed inconsistent correlations with quality indicators. These findings indicate that audience engagement alone may not be a reliable indicator of the clinical completeness or reliability of bladder cancer information.

### 3.7 Association between uploader type and BCWS

Across the 368 included hematuria videos, uploader categories comprised 104 urologists, 106 other healthcare professionals, 50 hospitals or medical institutions, 44 health science media sources, 42 non-professional uploaders, and 22 other/commercial/unclear sources. Unadjusted mean BCWS values ranged from 6.74 among other healthcare professionals to 7.77 among hospitals or medical institutions (Table 6). In the platform-adjusted model, uploader type was associated with BCWS overall (partial F = 4.26; df = 5,360). Compared with non-professional uploaders, videos uploaded by urologists had a lower adjusted mean BCWS (mean difference, −0.65; 95% CI, −1.17–−0.14), as did videos uploaded by other healthcare professionals (−0.88; 95% CI, −1.38–−0.39). Adjusted differences for hospitals or medical institutions, health science media, and other/commercial/unclear sources had 95% confidence intervals that included zero. These estimates describe completeness of the study-specific bladder cancer warning messages rather than overall clinical accuracy or causal effects of uploader identity.

**Table 6:** Uploader type and bladder cancer warning score in hematuria-related videos.

Uploader category	n	BCWS, mean ± SD	BCWS ≥7, *n* (%)	Adjusted mean difference (95% CI)
Non-professional uploader	42	7.21 ± 1.35	28 (66.7)	Reference
Urologist	104	7.14 ± 1.25	62 (59.6)	−0.65 (−1.17 to −0.14)
Other healthcare professional	106	6.74 ± 1.59	57 (53.8)	−0.88 (−1.38 to −0.39)
Hospital or medical institution	50	7.77 ± 1.35	36 (72.0)	−0.21 (−0.86 to 0.44)
Health science media	44	7.71 ± 1.14	33 (75.0)	−0.29 (−0.96 to 0.38)
Other/commercial/unclear	22	7.64 ± 1.42	18 (81.8)	0.20 (−0.50 to 0.89)

Adjusted mean differences were estimated in one multivariable linear regression containing uploader type and platform as categorical fixed effects. BCWS values are per-video means of three reviewer ratings. BCWS ≥7 is an exploratory study-specific adequacy threshold. CI, confidence interval; BCWS, bladder cancer warning score.

### 3.8 Association between uploader type and BC-PIS

Across the 363 included bladder cancer videos, uploader categories comprised 145 urologists, 55 other healthcare professionals, 71 hospitals or medical institutions, 33 health science media sources, 24 non-professional uploaders, and 35 other/commercial/unclear sources. Unadjusted mean BC-PIS values ranged from 5.21 among non-professional uploaders to 7.56 among hospitals or medical institutions (Table 7). After adjustment for platform, there was no clear overall association between uploader type and BC-PIS (partial F = 0.94; df = 5,355). Compared with non-professional uploaders, all uploader-category estimates had 95% confidence intervals that included zero. Platform-adjusted mean differences ranged from −0.54 (95% CI, −2.53–1.45) for other/commercial/unclear sources to 1.15 (95% CI, −0.87–3.17) for health science media. The estimates do not support uploader identity as an independent marker of BC-PIS-defined precision-immunotherapy completeness in this sample.

**Table 7:** Uploader type and bladder cancer precision immunotherapy score in bladder cancer-related videos.

Uploader category	n	BC-PIS, mean ± SD	BC-PIS ≥11, *n* (%)	Adjusted mean difference (95% CI)
Non-professional uploader	24	5.21 ± 4.75	4 (16.7)	Reference
Urologist	145	6.74 ± 4.39	29 (20.0)	0.75 (−0.98–2.48)
Other healthcare professional	55	7.42 ± 4.31	13 (23.6)	0.20 (−1.68–2.07)
Hospital or medical institution	71	7.56 ± 4.74	23 (32.4)	0.20 (−1.66–2.05)
Health science media	33	7.19 ± 5.76	12 (36.4)	1.15 (−0.87–3.17)
Other/commercial/unclear	35	5.31 ± 4.65	4 (11.4)	−0.54 (−2.53–1.45)

Adjusted mean differences were estimated in one multivariable linear regression containing uploader type and platform as categorical fixed effects. BC-PIS values are per-video means of three reviewer ratings derived from the supplied analysis files. BC-PIS ≥11 is an exploratory study-specific adequacy threshold. CI, confidence interval; BC-PIS, bladder cancer precision immunotherapy score.

### 3.9 Inter-rater reliability

Reliability analysis included all 368 hematuria videos and all 363 bladder cancer videos (Table 8). For BCWS, the overall ICC(2,1) was 0.693 (95% CI, 0.618–0.759), and Fleiss' kappa for BCWS ≥7 was 0.691 (95% CI, 0.627–0.750). Platform-specific BCWS ICCs were 0.993 for Bilibili, 0.374 for Douyin, and 0.542 for YouTube. For BC-PIS, the overall ICC(2,1) was 0.876 (95% CI, 0.841–0.905), and Fleiss' kappa for reviewer-specific BC-PIS ≥11 was 0.725 (95% CI, 0.659–0.784). Platform-specific BC-PIS ICCs were 0.988 for Bilibili, 0.783 for the calibrated Douyin reassessment, and 0.760 for YouTube; corresponding kappa values were 0.952, 0.598, and 0.683. These results show improved and acceptable reproducibility for the Douyin ratings, although threshold-level agreement remained lower than on Bilibili and YouTube.

**Table 8:** Inter-rater reliability of BCWS and BC-PIS, including calibrated douyin reassessment.

Module/platform	N	Rater 1 adequate	Rater 2 adequate	Rater 3 adequate	ICC(2,1) (95% CI)	Fleiss' kappa (95% CI)
BCWS/Overall	368	234/368	247/368	244/368	0.693 (0.618–0.759)	0.691 (0.627–0.750)
BCWS/Bilibili	120	52/120	48/120	48/120	0.993 (0.987–0.998)	0.954 (0.905–0.989)
BCWS/Douyin	99	79/99	79/99	81/99	0.374 (0.220–0.493)	0.357 (0.219–0.495)
BCWS/YouTube	149	103/149	120/149	115/149	0.542 (0.377–0.675)	0.478 (0.341–0.600)
BC-PIS/Overall	363	114/363	88/363	99/363	0.876 (0.841–0.905)	0.725 (0.659–0.784)
BC-PIS/Bilibili	124	8/124	7/124	7/124	0.988 (0.977–0.995)	0.952 (0.807–1.000)
BC-PIS/Douyin	90	52/90	35/90	38/90	0.783 (0.653–0.864)	0.598 (0.459–0.718)
BC-PIS/YouTube	149	54/149	46/149	54/149	0.760 (0.690–0.817)	0.683 (0.581–0.773)

Reviewer-specific adequate classifications were defined as BCWS ≥7 and BC-PIS ≥11. ICC(2,1) denotes a two-way random-effects, absolute-agreement, single-measure intraclass correlation coefficient. Confidence intervals are percentile intervals from 5,000 video-level nonparametric bootstrap resamples. All 368 hematuria and 363 bladder cancer videos were matched by platform-specific sequence number; no records were excluded or imputed. Douyin BC-PIS values reflect independent rescoring after structured rubric calibration. Primary BC-PIS results in Tables 4, 5 use the per-video mean of three reviewer scores. Background shading separates the BCWS and BC-PIS reliability results for faster comparison.

## 4 Discussion

This study examined bladder cancer-related social media information across two clinically connected domains: hematuria warning and precision immunotherapy. By analyzing videos from Bilibili, Douyin, and YouTube, we found that patient-facing information was abundant but uneven in quality, reliability, and clinical completeness. More importantly, the educational pathway from symptom recognition to pathology-based immunotherapy and precision treatment was only partially represented. This gap suggests that social media videos may provide fragmented pieces of bladder cancer information without consistently presenting the clinical logic that connects hematuria, diagnostic evaluation, pathological classification, and individualized treatment.

Hematuria-related videos showed substantial platform-level variation in their capacity to communicate bladder cancer warning information. YouTube and Douyin achieved higher BCWS scores than Bilibili, and adequate warning information was present in approximately four-fifths of Douyin and YouTube videos but only two-fifths of Bilibili videos. This difference is clinically meaningful because hematuria may serve as the first entry point into the bladder cancer diagnostic pathway. When videos fail to emphasize painless or gross hematuria, urological evaluation, or key diagnostic examinations, their value as early-warning educational materials is reduced. Even when overt misinformation is uncommon, insufficient diagnostic guidance may still produce an “omission risk,” whereby viewers recognize a symptom but do not receive enough information to understand the need for timely evaluation.

The pattern of content completeness further supports this concern. Across hematuria-related videos, basic concepts such as definition, symptoms, and etiology were generally covered more fully than diagnostic evaluation, treatment management, and follow-up. In other words, many videos were better at explaining what hematuria is than guiding viewers on what should be done next. For a symptom with both benign and malignant causes, this imbalance is important. Public-facing hematuria education should avoid unnecessary alarm, but it should also make clear that unexplained, painless, gross, or recurrent hematuria warrants clinical assessment. Without this escalation message, symptom education may remain informative but clinically incomplete.

Platform differences remained evident in the bladder cancer precision immunotherapy module after three-rater aggregation. Douyin had the highest mean BC-PIS (10.21), followed by YouTube (7.71) and Bilibili (3.28); however, only 33/90 (36.7%) Douyin videos met the ≥11 adequacy threshold. This indicates that the high average was driven by a subset of high-scoring videos rather than uniformly adequate information provision. This three-rater aggregated estimate is substantially less extreme than the previous single-score estimate and does not support a claim of uniform Douyin adequacy. The stronger Douyin performance may partly reflect its physician-dominated uploader composition and structured short-video format, whereas remaining score dispersion reflects variation in how completely individual videos connected pathology, staging, immunotherapy, and biomarker-informed treatment. More broadly, these findings should be considered in the context of persistent concerns regarding the quality and accuracy of health information on social media (11).

The calibrated independent reassessment materially strengthens the interpretation of the platform comparison. Douyin BC-PIS reliability improved to ICC 0.783 and kappa 0.598, indicating acceptable score reproducibility after calibration, but classification agreement remained imperfect. The primary estimate of 33/90 adequate Douyin videos should therefore still be interpreted with attention to the operational threshold. Prospective studies should predefine calibration procedures, retain reviewer-level scores, and validate the rubric in an external reviewer panel.

The most important content gaps in the bladder cancer module involved systemic immunotherapy and biomarker-informed precision treatment. Many videos, particularly on Bilibili and YouTube, did not adequately explain immune checkpoint inhibitors, PD-1/PD-L1, FGFR, biomarker testing, or individualized treatment selection. These omissions are clinically relevant. Contemporary bladder cancer care increasingly depends on pathological staging, risk stratification, immune-based therapy, and molecularly informed decision-making. Evidence supporting systemic immunotherapy and FGFR-directed therapy has reshaped treatment expectations in advanced urothelial carcinoma, making accurate patient-facing explanations increasingly important. Patient-facing videos that mention immunotherapy without explaining indications, disease stage, pathological context, or biomarker relevance may create unrealistic expectations or leave patients with an incomplete understanding of how treatment choices are made.

The required pathological and molecular context differs across these therapies. For intravesical BCG, histopathological confirmation after TURBT establishes urothelial carcinoma and defines stage, grade, concomitant carcinoma *in situ*, and recurrence or progression risk; these features distinguish patients with intermediate- or high-risk NMIBC who may benefit from BCG from those requiring surveillance, alternative intravesical treatment, or management for muscle-invasive disease (3). For PD-1/PD-L1-directed immune checkpoint inhibitors, pathology confirms the tumor type, while disease stage, treatment setting, prior platinum exposure or response, fitness for platinum-based chemotherapy, and, for selected indications, PD-L1 assay results determine whether and when treatment is appropriate; PD-L1 expression should therefore be presented as a context-dependent biomarker rather than a universal stand-alone test of benefit (4, 5). An updated review of PD-L1 immunohistochemistry in advanced urothelial bladder carcinoma further indicates that PD-L1 expression is associated with pathological grade and stage and may inform response prediction; however, spatial and temporal heterogeneity, differences among assays and scoring systems, and responses observed in some PD-L1-low or -negative tumors preclude its use as the sole determinant of immune checkpoint inhibitor eligibility (12). FGFR-directed therapy is different again: eligibility depends on demonstrating a susceptible activating FGFR alteration, commonly involving FGFR2 or FGFR3, with a validated molecular assay on adequate tumor material, in addition to confirming the relevant clinical setting and prior therapy (13). Thus, the term “precision immunotherapy” is educationally incomplete unless it explains how pathological diagnosis, stage and risk classification, treatment history, and molecular testing connect a named drug class to an appropriate patient population.

Another notable observation was the weak and inconsistent relationship between engagement metrics and information quality. Across the two modules, popularity indicators such as likes, comments, and views did not reliably correspond to higher DISCERN or GQS scores. This finding reinforces a common concern in digital health communication: algorithmic visibility and user engagement may reward accessibility, emotional appeal, or entertainment value rather than medical completeness. Previous evidence has shown that cancer misinformation and potentially harmful information can circulate widely on social media and may attract substantial engagement, highlighting the need to distinguish visibility from reliability (14). For cancer-related topics, this mismatch is particularly relevant because highly visible videos may shape patient perceptions even when they do not provide the most clinically useful information.

Compared with previous studies that primarily assessed the general quality of online health videos, this study places greater emphasis on clinical pathway completeness. Rather than treating hematuria and bladder cancer videos as separate information categories, we evaluated whether social media content connects the patient journey from hematuria recognition to diagnostic evaluation, pathology, BCG, immune checkpoint inhibitors, and precision treatment. This framework is especially relevant for bladder cancer because symptom recognition, pathological classification, and immune-based or biomarker-informed therapies are closely linked in real-world clinical decision-making. It also provides a more clinically oriented way to evaluate patient-facing digital content: not only whether information is accurate in isolated fragments, but whether it helps viewers understand the sequence of care from warning symptoms to treatment selection.

The AI-assisted structured extraction workflow also illustrates a practical approach to large-scale health information surveillance. Social media has become an important channel for health communication among the public, patients, and healthcare professionals, but its benefits are accompanied by concerns regarding reliability, interpretation, and unequal information quality (15). From an infodemiology perspective, structured analysis of online communication can help monitor the prevalence, distribution, and evolution of health-related information in digital environments (16). AI tools can help organize video-derived information, identify predefined clinical concepts, and improve the efficiency of structured content review. However, medical interpretation remains context-dependent and requires expert oversight. Concepts such as BCG, NMIBC, MIBC, immune checkpoint inhibitors, PD-1/PD-L1, and biomarker-informed treatment can be oversimplified or misclassified if evaluated without clinical judgment. Therefore, AI-assisted extraction should be regarded as a scalable support tool rather than a substitute for professional review, and final scoring should remain anchored in expert consensus. From an infodemiology and public health perspective, AI-assisted approaches may support large-scale monitoring of online health information, but misinformation management still requires information surveillance, health literacy improvement, quality control, and expert-guided knowledge translation (17, 18).

From a broader public health perspective, our findings suggest that improving online bladder cancer education requires both content-level and platform-level interventions. Clinicians and medical institutions should not only increase their participation in social media education but also present information in a pathway-based manner, linking hematuria warning, diagnostic evaluation, pathology, staging, and treatment options. At the platform level, mechanisms that promote credible, complete, and professionally reviewed cancer information may help reduce the mismatch between popularity and educational value. Systematic reviews of online health misinformation and public health recommendations have emphasized that misinformation is not only a content problem but also a communication, literacy, and platform-governance problem (19, 20). Therefore, improving social media-based bladder cancer education will require coordinated efforts from clinicians, institutions, platform operators, and digital health researchers.

In the bladder cancer module, the apparent unadjusted differences between uploader groups were attenuated after platform adjustment, and no uploader category showed a clear independent association with BC-PIS. This pattern indicates that creator composition and platform were strongly intertwined: for example, most Douyin videos were uploaded by clinicians, whereas institutional and health-media sources were concentrated on YouTube. Accordingly, professional or institutional identity should not be used alone as a quality signal for precision-immunotherapy education. Platform-specific presentation practices and explicit coverage of pathology, stage, intravesical therapy, systemic immunotherapy, and biomarkers may be more actionable targets than uploader credentials alone.

Uploader identity alone may not fully reflect educational completeness in online bladder cancer information. After adjustment for platform, urologist and other-healthcare-professional videos had lower BCWS values than non-professional uploader videos, whereas estimates for institutional and health-media sources were imprecise and included no clear difference. This finding should not be interpreted as evidence that professional content is generally less accurate, as BCWS specifically evaluates the inclusion of bladder cancer warning and diagnostic-escalation messages; clinically accurate videos focused on other aspects of hematuria may therefore receive lower scores. Professional credentials may reflect clinical expertise; however, completeness of patient-facing communication may depend on whether key diagnostic and treatment information is explicitly addressed. Structured content guidance may therefore represent a potential strategy for improving the completeness of patient-facing information across uploader groups.

These interpretations should remain within the study's descriptive scope. The cross-sectional analysis demonstrates differences in measured content characteristics among the sampled videos and identifies omissions relative to the prespecified scoring framework. It did not measure exposure to the videos, establish that viewers understood or acted on their content, or test effects on health-seeking behavior, treatment expectations, decision-making, or clinical outcomes. Accordingly, the observed platform-level differences and content gaps should not be interpreted as evidence that any platform or video caused benefit or harm to patients; such effects require prospective audience-based or outcome-based studies.

Although BCWS and BC-PIS showed acceptable inter-rater reproducibility in this study, they should be considered exploratory content assessment frameworks rather than validated measurement instruments. Further work is needed to establish their validity and broader applicability. Future studies should first evaluate content validity through multidisciplinary expert assessment, potentially using Delphi-based consensus approaches and Content Validity Index evaluation to determine whether the selected domains adequately represent clinically relevant patient-facing information (21). Additional studies should examine construct validity by assessing the relationships between BCWS/BC-PIS scores and established measures of health information quality, expert evaluations, or other indicators of educational completeness (22). External validation in independent datasets, platforms, and reviewer groups will be needed to assess the generalizability of these scoring systems. The current adequacy thresholds (BCWS ≥7 and BC-PIS ≥11) were pragmatic, study-specific choices and should be further calibrated in larger samples and evaluated against clinically meaningful outcomes. Until such validation is completed, BCWS and BC-PIS should be interpreted as structured tools for comparative analysis of online bladder cancer information rather than universal measures of educational quality.

## 5 Limitation

This study has several limitations. First, it was cross-sectional and reflected platform content at a single time point; search results, algorithms, and video metrics may change over time. Second, only one predefined keyword was used for each module, which may have limited the comprehensiveness of retrieved videos, as patients may use different expressions or alternative terms when searching for bladder cancer-related information. Therefore, the included videos should be interpreted as a predefined search snapshot rather than a complete representation of all available online educational content. In addition, the number of included videos varied across platforms, with fewer Douyin videos retrieved under the predefined search criteria. Third, platform-specific data availability differed; for example, YouTube did not provide favorites or shares, and Douyin views were unavailable. Fourth, although predefined criteria were used and final scores were determined by consensus among three urologists, scoring tools such as DISCERN, PEMAT, GQS, BCWS, and BC-PIS still involve subjective judgment. Fifth, AI tools were used only to support preliminary structured content extraction; this study did not compare the performance of different AI models or calculate AI-human agreement. Sixth, the high and concentrated scores for Douyin videos, especially in the bladder cancer module, should be interpreted cautiously. In addition, although multiple comparisons were adjusted using Bonferroni correction, the exploratory nature of cross-platform analyses should still be considered when interpreting statistical differences. Finally, this study assessed video content rather than viewer comprehension, health-seeking behavior, treatment decision-making, or clinical outcomes.

The uploader analyses also have specific limitations. Uploader categories were assigned from public profiles and were consolidated because several original categories and uploader-by-platform cells were sparse; residual misclassification and limited precision are therefore possible. Sparse or empty cells, especially in the Douyin bladder cancer sample, precluded reliable uploader-by-platform interaction analyses. Platform adjustment addresses measured platform differences but does not eliminate confounding by topic, intended audience, production resources, video duration, or other unmeasured characteristics. The cross-sectional associations cannot establish that uploader identity caused the observed score differences.

BCWS and BC-PIS were newly developed, study-specific content indices. Their domains were grounded in clinical guidance and key therapeutic evidence (2–6), but formal content-validity indices, construct validity, criterion validity, test-retest reliability, and external validation were not assessed. Independent reviewer-level analyses demonstrated acceptable overall reproducibility, although BCWS agreement was lower on Douyin and YouTube and BC-PIS threshold agreement after Douyin calibration remained moderate rather than perfect. The equal weights and the ≥7 and ≥11 thresholds remain prespecified pragmatic choices rather than empirically optimized cutoffs. Accordingly, the platform comparisons and adequacy rates should be interpreted as exploratory until BCWS and BC-PIS undergo further validation in independent settings.

## 6 Conclusion

Social media videos on hematuria and bladder cancer provide accessible health information but incompletely connect early warning symptoms with modern bladder cancer diagnosis and treatment pathways. Uploader identity was not a consistent independent marker of educational completeness: selected professional uploader groups had lower BCWS-defined warning completeness than non-professional uploaders after platform adjustment, while uploader type was not clearly associated with BC-PIS in the bladder cancer module. These findings should not be interpreted as indicating lower overall accuracy of professional content. Rather, they suggest that structured, pathway-based content guidance may improve the completeness of patient-facing information across uploader groups. The conclusions are limited to the sampled content and study-specific scores and do not establish effects on patient comprehension, behavior, decisions, or clinical outcomes.

## Data Availability

The original contributions presented in the study are included in the article/Supplementary Material, further inquiries can be directed to the corresponding authors.
